# A 3-D Full Convolution Electromagnetic Reconstruction Neural Network (3-D FCERNN) for Fast Super-Resolution Electromagnetic Inversion of Human Brain

**DOI:** 10.3390/diagnostics12112786

**Published:** 2022-11-14

**Authors:** Yu Cheng, Li-Ye Xiao, Le-Yi Zhao, Ronghan Hong, Qing Huo Liu

**Affiliations:** 1 Institute of Electromagnetics and Acoustics, School of Electronic Science and Engineering, Xiamen University, Xiamen 361005, China; 2 Fujian Provincial Key Laboratory of Electromagnetic Wave Science and Detection Technology, Xiamen University, Xiamen 361005, China; 3 Department of Electrical and Computer Engineering, Duke University, Durham, NC 27708, USA

**Keywords:** human brain imaging, super-resolution, deep learning, high contrast, electromagnetic inversion

## Abstract

Three-dimensional (3-D) super-resolution microwave imaging of human brain is a typical electromagnetic (EM) inverse scattering problem with high contrast. It is a challenge for the traditional schemes based on deterministic or stochastic inversion methods to obtain high contrast and high resolution, and they require huge computational time. In this work, a dual-module 3-D EM inversion scheme based on deep neural network is proposed. The proposed scheme can solve the inverse scattering problems with high contrast and super-resolution in real time and reduce a huge computational cost. In the EM inversion module, a 3-D full convolution EM reconstruction neural network (3-D FCERNN) is proposed to nonlinearly map the measured scattered field to a preliminary image of 3-D electrical parameter distribution of the human brain. The proposed 3-D FCERNN is completely composed of convolution layers, which can greatly save training cost and improve model generalization compared with fully connected networks. Then, the image enhancement module employs a U-Net to further improve the imaging quality from the results of 3-D FCERNN. In addition, a dataset generation strategy based on the human brain features is proposed, which can solve the difficulty of human brain dataset collection and high training cost. The proposed scheme has been confirmed to be effective and accurate in reconstructing the distribution of 3-D super-resolution electrical parameters distribution of human brain through noise-free and noisy examples, while the traditional EM inversion method is difficult to converge in the case of high contrast and strong scatterers. Compared with our previous work, the training of FCERNN is faster and can significantly decrease computational resources.

## 1. Introduction

Electromagnetic (EM) inversion is a nondestructive technique that, through analyzing the scattering field under the given illumination in the domain of interest (DOI) [1], obtains electromagnetic properties such as permittivity, conductivity and permeability of the unknown scatterer located in the inaccessible region. It has a wide range of applications in medical imaging [2,3], remote sensing [4], geophysical exploration [5,6], microwave imaging [7,8] and so on.

In general, inverse scattering methods can be classified into noniterative and iterative methods. In the circumstance of weak scattering, noniterative methods are usually used [9,10,11], and the inverse problem can be expressed as a linearized equation to obtain an approximate solution. Noniterative methods can provide fast reconstruction results with guaranteed accuracy, especially in tasks with strong scattering and high contrast.

The reconstruction of unknown scatterers by the iterative methods [12,13,14,15,16,17] for nonlinear problems is achieved through iteratively minimizing an objective function that quantifies the mismatch between the calculated and measured scattering field data. However, iterative methods take a long time and high computational costs in their reconstruction process, thus they are usually not suitable for real-time inversion.

In recent years, with the rapid development of machine learning, the method of solving the electromagnetic inverse scattering problem based on machine learning has become a research hotspot. Pixel-based machine learning EM inversion methods are proposed and can achieve better results [18,19,20,21]. The semi-join extreme learning machine (SJ-ELM) [22] proposed by our group in previous work can achieve super-resolution three-dimensional (3-D) microwave imaging of objects with high contrasts. The 3-D objects are divided into multiple 1-D data sequences, and each sequence corresponds to a sub-model, however, for the scatterers with complex structures and large electrical dimension, it will be invalid.

As the most complex and important organ in the organisms, the human brain is significant interest for imaging [23]. Up to now, many microwave imaging methods have been proposed for human brain. In [24], Saeed et al. works on brain cancer and applies a statistical model to test and discuss brain tumor images. In [25], three nonlinear iterative reconstruction algorithms, i.e., contrast source inversion (CSI), subspace-based optimization method (SOM) and distorted born iterative method (DBIM), are employed for brain stroke imaging. In [26], the inversion of *S*-parameter data collected in a metallic chamber is performed with a nonlinear inversion strategy in Lebesgue spaces with nonconstant exponents. Coli et al. [27] employ massive parallel computation from domain decomposition method and regularization techniques for the early detection and monitoring of brain strokes. In [28], a multistatic system prototype for brain stroke detection is proposed.

However, because of the high contrast of brain tissue electrical parameters, super-resolution 3-D human brain imaging is a strong nonlinear electromagnetic inverse scattering problem; it is not only difficult to reconstruct, but also requires a lot of computer memory and operation time for these methods. In [29], AHCN-LNQ (adaptive histogram contrast normalization with learning-based neural quantization) is proposed to initially perceive a brain tumor in a person with early signs of brain tumor. A hybrid neural network electromagnetic inversion scheme (HNNEMIS) [30] proposed by our group in previous work can achieve super-resolution 3-D human brain imaging. In [30], the semi-join back propagation neural network (SJ-BPNN) is proposed to map the relationship between scattering fields and electrical parameters distribution of human brain, it discretized 3-D objects into multiple 1-D data sequences, and each sequence corresponds to a BPNN; thus, a huge number of models need to be trained. Therefore, it still requires a large memory cost for imaging.

Thus, how to achieve 3-D super-resolution human brain imaging with less computational cost is still a challenging task. This work proposes a new EM inversion scheme based on deep neural network for super-resolution 3-D microwave imaging of human brain. The scheme can be divided into EM reconstruction module and image optimization module. For the EM reconstruction module, a 3-D full convolution electromagnetic reconstruction neural network (3-D FCERNN) is proposed to reconstruct 3-D permittivity and conductivity distribution of human brain from the measured scattered electric field data. Then, for the image optimization module, U-Net is further employed to enhance the image quality and anti-noise ability of the whole scheme for human brain imaging. In order to make the training cost low, a training strategy based on the human brain characteristics is also introduced to create training dataset. 

The main contributions of this work can be summarized as follows: (a) This work proposes a 3-D FCERNN, which consists entirely of convolutional layers, to improve the accuracy and reduce the huge computation cost for super resolution 3-D inversion. The training parameters is far less than the network which used fully connected layers. The FCERNN can reduce the computation burden, and can effectively alleviate the phenomenon of over fitting, and improve the convergence performance of the network. (b) For the super-resolution imaging problems of 3-D scatterers, how to minimize the training cost and training time has always been a challenging task. Our proposed inversion network can directly reconstruct 3-D objects, which greatly reduces the number of training models, and effectively shortens the training time and minimizes the training cost. Compared with HNNEMIS, the training time of our scheme is greatly reduced, based on the conduction of 3-D numerical example with 512 × 512 × 512 voxels. (c) A dual module inversion scheme is proposed for 3-D super resolution inversion of human brain, where the first module, FCERNN, has strong nonlinear mapping capability to map the scattered field to 3-D electrical parameters. The second module, U-Net, is used to enhance the inversion quality of the output of the previous module. The second module has not only strong nonlinear mapping ability, but also has strong edge information analysis ability, which can calibrate the details of the results to make the inversion results more accurate. 

This work is organized as follows. In Section 2, the formulation of mixed FEM is briefly reviewed, and the proposed 3-D FCERNN and inversion scheme are discussed in detail. In Section 3, normal human brain and human brain with an abnormal scatterer are tested to illustrate the feasibility of the proposed method. In Section 4, some traditional inversion schemes and machine learning based brain reconstruction schemes are discussed and analyzed. Finally, we summarize this work in Section 5.

## 2. Materials and Methods

### 2.1. Mixed FEM for Forward Simulation

In this work, the mixed finite element method (mixed FEM) [31] is used to generate training and test samples. The mixed FEM can stably and accurately perform biological electromagnetic human brain simulations in the microwave frequency band by applying Gauss’ law as the constraint condition. 

Since the magnetic susceptibility of human brain and air is weak, the permeability is assumed as a constant $\mu_{0}$. Using the implied time convention $e^{j\omega t}$, the Helmholtz equation of the scattered magnetic field $H^{s}$ can be obtained as follows:

(1) $$\nabla \times \left({\overset{=}{\varepsilon }}_{r}^{-1}\nabla \times H^{s}\right)-k_{0}^{2}{\overset{=}{\mu }}_{r}H^{s}=j\omega \varepsilon_{0}\nabla \times \left(I-{\overset{=}{\varepsilon }}_{rb}{\overset{=}{\varepsilon }}_{r}^{-1}\right)E^{i}+k_{0}^{2}\left({\overset{=}{\mu }}_{r}-{\overset{=}{\mu }}_{rb}\right)H^{i}$$

(2) $$k_{0}^{2}\nabla \cdot \left[\left({\overset{=}{\mu }}_{r}-{\overset{=}{\mu }}_{rb}\right)H^{i}\;\right]=-k_{0}^{2}\nabla \cdot \left({\overset{=}{\mu }}_{r}H^{s}\right)$$

where ${\overset{=}{\varepsilon }}_{rb}$ and ${\overset{=}{\mu }}_{rb}$ are the relative complex permittivity tensor and relative complex permeability tensor of the background medium, respectively, ${\overset{=}{\varepsilon }}_{r}$ and ${\overset{=}{\mu }}_{r}$ are the complex relative permittivity and complex relative permeability of the materials, respectively, $E^{i}$ and $H^{i}$ are the incident electric and magnetic fields in a homogeneous background medium (air or a matching fluid) which can be computed directly by analytical solutions, $k_{0}$ and $\varepsilon_{0}$ are the wavenumber and permittivity of vacuum, and ω is the angular frequency. ${\tilde{\varepsilon }}_{r}$ can also be further represented by the relative permittivity and conductivity:

(3) $${\tilde{\varepsilon }}_{r}=\varepsilon_{r}+\sigma /\left(j\omega \varepsilon_{0}\right)$$

where $\varepsilon_{r}$ is the relative permittivity and σ is the conductivity. Ref. [31] presents more detailed description of the mixed FEM in solving bio-electromagnetic problems including brain electromagnetic simulations.

### 2.2. Proposed Deep Learning Inversion Scheme

**Figure 1:**
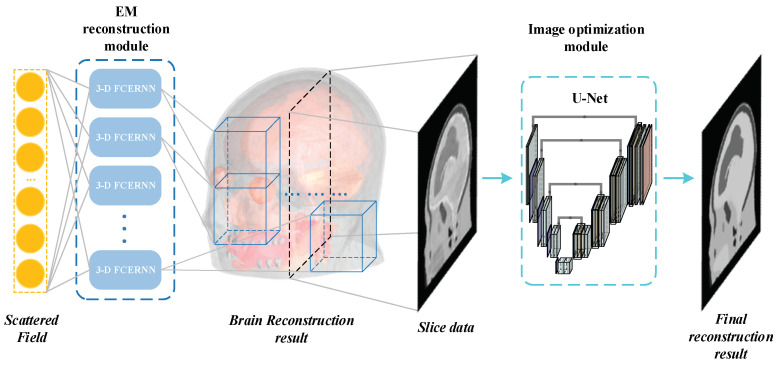
The algorithm flow of the scheme.

This section introduces the proposed human brain training dataset generation strategy, then describes the proposed 3-D FCERNN in detail, followed by the U-Net for the image optimization module. The proposed scheme can be divided into electromagnetic reconstruction module and image optimization module. The algorithm flow of the scheme is shown in Figure 1, where the 3-D FCERNN is proposed to reconstruct the measured scattered electric field or magnetic field into 3-D human brain electrical characteristic data. Then, in the image optimization module, the 3-D human brain images generated by the reconstruction module are cut into 2-D images by *XY* slices and input into U-Net for optimization.

#### 2.2.1. Training Dataset Building Strategy

For the inversion method based on deep neural network, whether the training dataset can be constructed scientifically and effectively directly determines the training performance of the machine learning model. How to make the training dataset close to the real brain and require only a small number of samples is a big challenge for 3-D super-resolution brain imaging. Obviously, the traditional training dataset generation strategy, where the structure, size, location, and electrical properties of scatterers are randomly set in the imaging domain, is not economic for the human brain problem, because millions of training samples will be needed. To address this point, a new training set building strategy based on the changes of brain characteristics is proposed to generate dataset. First, a basic brain model provided by NEVA electromagnetics [32] is constructed, and then the corresponding electrical characteristics of each tissue at 300 MHz are obtained through [33]. Secondly, for different training cases, the human brain model and internal structure are obtained by scaling the basic model randomly within the range of 0.8 to 1.2 (with the step of 0.1) according to the value in [30]. Thirdly, since there are individual differences among different brain tissues. The boundary of each tissue needs to be deformed. In this operation, each point on each tissue boundary is displaced by a normal distribution in the *x*, *y* and *z* directions, respectively. To ensure the universality of the case, all brain tissues are deformed with a random order.

**Figure 2:**
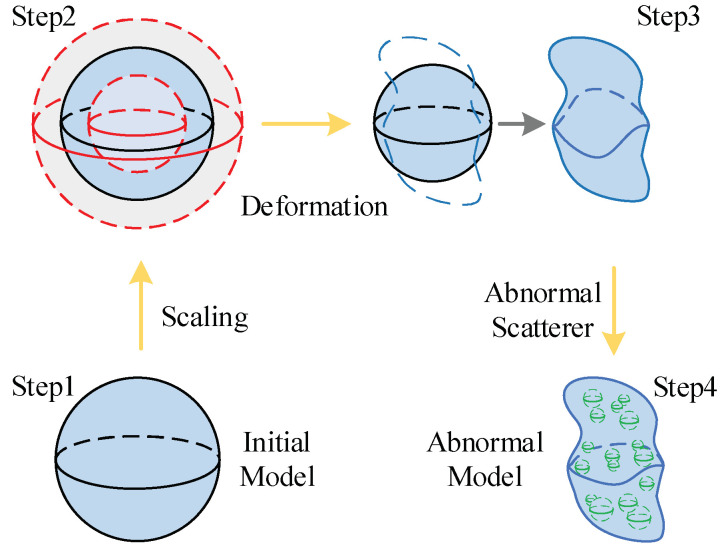
Training data set generation strategy based on human brain characteristics.

Considering the task of detecting abnormal conditions such as lesions, bleeding or tumors in human brain, abnormal scatterers of different sizes and electrical properties are randomly added into human brain. In this work, a spherical anomalous scatterer is added to the training samples. The relative permittivity of the anomalously scatterer is randomly selected from 90, 120 and 150, while the conductivity is randomly selected from 1.038 S/m, 1.3840 S/m and 1.73 S/m, and the radius is randomly selected from 4 mm, 7 mm and 10 mm. Meanwhile, to evaluate the performance for solving the high contrast inversion problem. The abnormal scatterer with high contrast is selected. To easily demonstrate this process, a regular sphere is taken as an example (Figure 2). Step 1: A generic human brain is selected as the basic model; Step 2: Scale the basic model within a ratio, which is randomly in the range from 0.8 to 1.2, including the size and the electrical properties, to ensure the validity and universality of the training dataset; Step 3: Deform each tissue to guarantee the individual differences in different human brains, and the degree of deformation follows the normal distribution; Step 4: Randomly add abnormal scatterers with different sizes and electrical properties in the human brain as abnormal conditions such as lesions, bleeding or tumors.

#### 2.2.2. 3-D FCERNN

**Figure 3:**
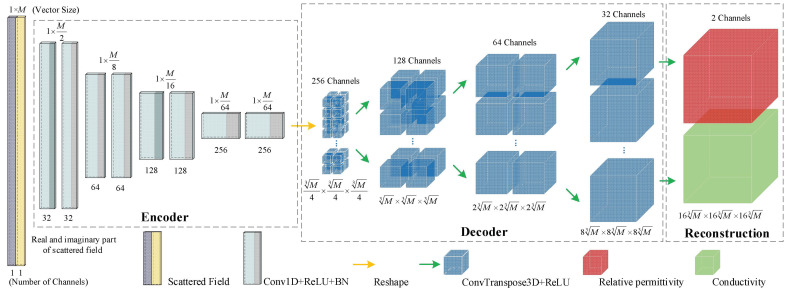
Structure of the proposed 3-D FCERNN.

In order to quickly and accurately image the electrical properties of human brain from the measured scattered fields, a full-convolutional deep neural network, i.e., 3-D FCERNN (Figure 3), is proposed in this work. The network takes the scattered electrical field data as input and outputs the relative permittivity and conductivity distribution of imaging domain.

Compared with the SJ-BPNN used in the previous work [30], 3-D FCERNN structure is more complex, and can solve a task with higher nonlinearity. Moreover, the training parameters are far less than the network with fully connected layers. FCERNN can reduce the computation burden, effectively alleviate the phenomenon of over-fitting and reduce the training time.

The 3-D FCERNN consists of three modules, namely an encoder module, a decoder module and a reconstruction module. The encoder module is used to receive the scattered field data and extract its internal information to obtain the corresponding 3-D feature map; the decoder module is used to restore the obtained feature map to a certain size of the initial reconstruction data information; and the reconstruction module is used to reconstruct the initial electrical properties into the final reconstruction data of the required size.

For the *j*th training sample, the input is a 2-channel column vector $x_{j}={\left[[r_{1j},r_{2j},\cdots r_{Mj}{]}^{T},[i_{1j},i_{2j},\cdots i_{Mj}\right]}^{T}{]}^{T}\in R^{2\times M}$, representing the real ($r_{Mj}$) and imaginary ($i_{Mj}$) parts of the scattered field obtained by the combination of all transmitters and receivers, respectively, where M is the dimension of each channel of data input during each training. Meanwhile, the corresponding output of the *j*th training sample is discretized into $N_{1}\times N_{2}\times N_{3}$ voxels. Therefore, the output of the *j*th training sample can be expressed as a 3-D matrix $P_{j}\in R^{N_{1}\times N_{2}\times N_{3}}$.

For the super-resolution imaging tasks such as human brain imaging, $N_{1}\times N_{2}\times N_{3}$ is an extremely large number, requiring a large amount of computer memory. Therefore, in the inversion scheme proposed in this work, the complete 3-D output data is evenly divided into *N* subset blocks, and each output of 3-D FCERNN model corresponds to a subset block. Thus, the complete data can be expressed as $P_{j}=p_{j}^{1}\cup^{\;}\cdots p_{j}^{n}\cdots \cup^{\;}p_{j}^{N}$, where $p_{j}^{n}\in R^{\frac{N_{1}}{N}\times \frac{N_{2}}{N}\times \frac{N_{3}}{N}}$.

The encoder module of 3-D FCERNN is all composed of convolution layers. The input scattered field dataset is a double-channel column vector of 2 × *M*; the convolution kernel sizes of the eight convolution layers in Figure 3. are 2, 2, 2, 2, 2, 4 and 1, respectively, and the number of kernels is 32, 32, 64, 64, 128, 128, 256 and 256. In the first, third, fifth and seventh convolution layers, we use convolution stride 2, 4, 2 and 4 to replace the pooling layer, because stridden convolution can be fully differentiable and allow the network to learn its own down-sampling while keeping more details. In addition, we carry out Batch Norm (BN) [34] operation on the output of each convolution layer, and BN layer can normalize the feature map of batches to avoid the instability of network performance caused by excessive data. The calculation formula of BN layer is as follows:

(4) $${\hat{y}}_{i}={\mathrm{BN}}_{\gamma ,\beta }\left(y_{i}\right)=\gamma \frac{y_{i}-\mu_{\beta }}{\sqrt{\sigma_{\beta }^{2}+\chi }}+\beta$$

where $y_{i}$ and ${\hat{y}}_{i}$ are the input and output of BN layer, respectively; $\mu_{\beta }$ is the mean value of $y_{i}$, $\sigma_{\beta }$ is the standard deviation of $y_{i}$, γ is the scaling factor, β is the translation bias, χ is a constant (0.001 by default). The encoder module eventually processes the scattered field data into a 256-channel column vector of ($\frac{M}{64}$ × 1) × 256, which is then transformed by the Reshape layer into a 4-D feature map of size $\frac{\sqrt[{3}]{M}}{4}\;$×$\;\frac{\sqrt[{3}]{M}}{4}\;$×$\;\frac{\sqrt[{3}]{M}}{4}\;$× 256.

The decoder module is composed of three deconvolution layers, and the number of convolution kernels is 128, 64 and 32, respectively. Because deconvolution can inversely map the eigenvalue back to the input data space, a deeper relationship between the feature graph and the input graph can be obtained. Compared with the combination of up-sampling layer and convolution layer, the deconvolution layer can better consider the details of reconstructed data under the condition of a smaller number of parameters, and reduce the training time and memory consumption.

The final reconstruction module is composed of a 3-D deconvolution layer with a convolution kernel number of 2. The initial reconstruction data information generated by the decoder module is reconstructed into the required size of the 2-channel final reconstruction data, namely the relative dielectric constant and conductivity distribution in DOI. The ReLU activation function is expressed below

(5) $$f_{ReLU}\left(x\right)=\max\left(0,x\right)$$

Meanwhile, it should be noted that each sub-model is independent of each other, so there is no information exchange between the sub-models. For fully connected neural networks such as BP and ELM, with the increase in their output layer, the nonlinearity of tasks will be enhanced. In order for the results to converge, the number of nodes in the hidden layer will be greatly increased, and all nodes of each layer need to be connected to all nodes of the two layers before and after, resulting in a sharp increase in the weight parameters that need to be trained, which means expensive computational cost and hard neural network convergence. At the same time, the generalization of the model will become worse.

Therefore, compared with the fully connected network strategy, 3-D FCERNN based on full convolution is more economical, has better generalization ability and shorter training time for super-resolution 3-D inversion problems.

#### 2.2.3. U-Net

**Figure 4:**
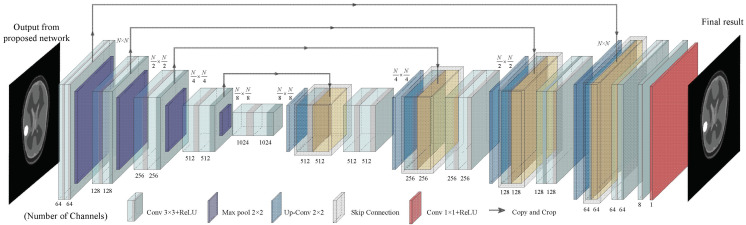
The structure of U-Net.

In order to further improve the imaging performance of FCERNN, this work employs U-Net as the imaging optimization module. Its input is the 2-D slices of the results from 3-D FCERNN and the corresponding output is the improved inversion results. U-Net was first proposed for biomedical image segmentation [35]. A deep network with a U-shaped symmetrical structure, it is composed of a down-sampling part and an up-sampling part (Figure 4). The down-sampling part is the classical convolutional neural network structure. It contains four conv-pool layers, and each conv-pool layer is composed of two 3 × 3 convolution layers, two rectifying linear unit (ReLU) and a 2 × 2 maximum pooling layer, which can obtain segment maps at pixel level. The up-sampling part consists of four up-sampling layers. Each up-sampling layer is similar to a conv-pool layer. In other words, 3 × 3 up-convolution is used to replace the 2 × 2 maximum pooling in conv-pool layer so that the feature map can maintain uniform color difference. Global feature information and local feature details are extracted from the input image through the down-sampling part, and then the segment map is restored to the input size from the up-sampling part. In addition, the feature maps obtained by each convolution layer of U-Net will be added to the corresponding up-sampling layer through skip-concatenate, so the feature maps of each layer will be effectively used in subsequent calculations to improve the accuracy of the model results.

## 3. Results

In this section, two numerical cases are presented to demonstrate the effectiveness of the deep neural network-based inversion scheme for the super-resolution 3-D human brain imaging. The first numerical case is used to evaluate the performance of the proposed model for the normal brain. The second case is used to evaluate the performance of the model in the detection of the anomalous scattering in the human brain. White Gaussian noise pollution with different signal-to-noise ratio (SNR) is added to the data in two cases to test the anti-noise ability of the proposed model.

### 3.1. Electromagnetic Inversion Simulation Experiment Settings

Since it is difficult to obtain a large amount of real human brain scattered field data, EM simulation is used to obtain human brain scattered field data as the training data in this work. The operating frequency of the two numerical cases is set at 300 MHz, and the corresponding wavelength in air is $\lambda_{0}$ = 1 m. DOI is a cube with a side length of 0.24 m, and the center of DOI is the origin of the coordinate axis. The transmitters and receivers (modeled as point electric dipoles) surround the DOI’s human brain model in four layers: the first layer has 32 transmitters/receivers placed in a 0.12 m radius circle with a step of 11.25° at *z* = −0.06 plane; the second layer has 16 transmitters/receivers placed in a 0.115 m radius circle with a step of 22.5° at *z* = 0 plane; the third layer has 8 transmitters/receivers placed in a 0.1 m radius circle with a step of 45° at *z* = 0.06 plane; and the fourth layer has 4 transmitters/receivers placed in a 0.06 m radius circle with a step of 90° at z = 0.1 plane. There are 60 transmitters/receivers in this setup so that each receiver will receive signals from 59 other transmitters. That is, a total of 3 × 60 × 59 = 10,620 measured scattered electric field data with *x*, *y* and *z* components are received. In order to reduce the number of training parameters and the dependence of scattered field data and improve the generalization of the model, this work sums the signals of 59 other transmitters received by 60 receivers, respectively, and obtains 60 × 3 = 180 measured values, and then take it as input to 3-D FCERNN, that is, the input size is equal to 2 × *M* = 360. The virtual human brain simulation model was constructed by NEVA Electromagnetism. All the forward and inverse computations are performed on a workstation with 20-cores CPU, 256 GB RAM and the NVIDIA Geforce RTX 3090 GPU.

### 3.2. Setups of Training Sample and Network

**Figure 5:**
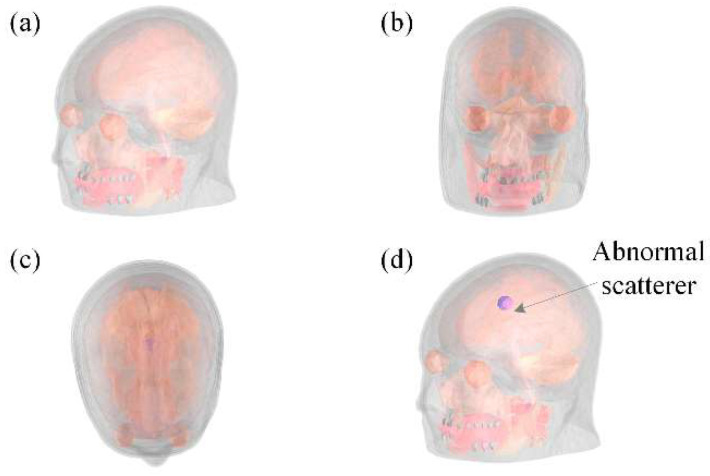
Two numerical cases used in this work: (**a**) squint view of the normal brain model; (**b**) frontal view of the normal human brain model; (**c**) top view of the normal human brain model; (**d**) oblique view of the human brain model with anomalous scatterers.

The imaging domain is represented as a cube box of 0.24 × 0.24 × 0.24 m^3^ (Figure 5). In the first case, the reconstructed subjects were normal human brains. The imaging domain is discretized into 256 × 256 × 256 voxels, and the size of each voxel was 0.94 × 0.94 × 0.94 mm^3^ (or 9.4 × 10^−4^ λ × 9.4 × 10^−4^ λ × 9.4 × 10^−4^ λ). In this example, 190 groups of samples are used to train the 3-D FCERNN model. In the second case, the brains with abnormalities such as tumors are performed. To test the performance of the proposed scheme in the super-resolution reconstruction task, we discretized the imaging domain into 512 × 512 × 512 voxels to challenge the higher resolution. The size of each voxel was 0.47 × 0.47 × 0.47 mm^3^ (or 4.7 × 10^−4^ λ × 4.7 × 10^−4^ λ × 4.7 × 10^−4^ λ). In this example, 250 groups of samples are used to train the 3-D FCERNN model. Due to the large number of discrete voxels in the two examples, direct reconstruction of all the voxels will require huge computing resources and increase the training time. Therefore, the whole imaging domain voxels are divided into several cube boxes, and each cube box is reconstructed by an FCERNN. 

In order to evaluate the imaging performance of the method, the model misfit and data misfit under L2 norm are defined as follows:

(6) $${\mathrm{Misfit}}_{\mathrm{model}}=\frac{{∥{\mathrm{Mp}}_{R}-{\mathrm{Mp}}_{T}∥}_{2}}{{∥{\mathrm{Mp}}_{T}∥}_{2}}$$

(7) $${\mathrm{Misfit}}_{\mathrm{data}}=\frac{{∥{\mathrm{Dp}}_{R}-{\mathrm{Dp}}_{T}∥}_{2}}{{∥{\mathrm{Dp}}_{T}∥}_{2}}$$

where ${\mathrm{Mp}}_{T}$ and ${\mathrm{Mp}}_{R}$ are the true electrical parameters of the model and their reconstructed values for all voxels, respectively.${\;\mathrm{Dp}}_{T}$ and ${\mathrm{Dp}}_{R}$ are the measured scattered field received by all receivers and the scattered field vector obtained from the reconstructed result, respectively.

In this work, the constructed human brain model contains 16 different tissues, and the corresponding electrical characteristics of each tissue at 300 MHz are listed in [30]. It is not difficult to find that the electrical characteristics of brain tissues at this frequency are much greater than that of air, and there are great differences among tissues, so super-resolution imaging of human brain is a high-contrast EM inverse problem. The training samples are obtained by the proposed training set construction strategy based on the changes of human brain characteristics. Based on the basic human brain parameters, the electrical properties of each human brain tissue are multiplied by a random multiplier between 0.8 and 1.2. For the second example, anomalous scatterers with radii of 4 mm, 7 mm, and 10 mm are randomly distributed in the human brain. Their relative permittivity is randomly set to 90, 120, and 150, and the conductivity is randomly set to 1.038 S/m, 1.384 S/m, and 1.73 S/m.

In addition, in order to make 3-D FCERNN converge rapidly, this work adopts the mean square error (MSE) as the loss function of the model, and the expression of MSE loss function is as follows

(8) $${\mathrm{MSE}}_{\mathrm{loss}}=\frac{1}{m}\overset{m}{\underset{i=1}{\sum }}{\left(y_{i}-{\hat{y}}_{i}\right)}^{2}$$

where, *m* is the number of samples, $y_{i}$ is the model reconstruction electrical parameter, ${\hat{y}}_{i}$ is the real electrical parameter, it should be noted that both $y_{i}$ and ${\hat{y}}_{i}$ are normalized.

### 3.3. Case I: Normal Human Brain

In order to evaluate the imaging performance of the proposed deep neural network-based electromagnetic inversion method for normal human brain, a normal human brain model never appeared in the training data set is selected for testing. The differences between the test set and the averaged training mode are shown in Table 1. In this case, both 3-D FCERNN and U-Net are GPU accelerated. During the training process, the 3-D FCERNN required approximately 3 h of training on a computer with 24 GB of video memory, and the run time is reduced to 1 min in testing process. Meanwhile, the whole human brain reconstructed by 3-D FCERNN is sliced into U-Net for optimization, and the training time and test time of U-Net is about 80 min and 1 min, respectively.

**Figure 6:**
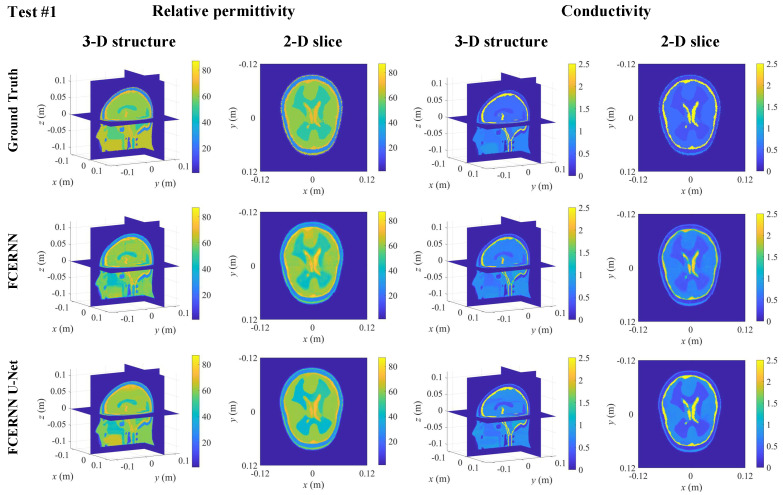
The reconstructed result obtained from FCERNN and U-Net optimization.

The first row of Figure 6 shows the true electrical parameters distribution. The second row shows the 3-D slices of human brain reconstructed by 3-D FCERNN and the corresponding 2-D slice at *z* = 0.02 m plane, where the first two columns are relative permittivity distributions and the last two columns are conductivity distributions. It can be seen that the initial reconstruction results obtained by 3-D FCERNN can accurately reconstruct the position, shape and size of each human brain tissues, but there are some noises and inaccuracies at the boundaries of tissues, and the electrical parameters of tissues are not accurate enough. Fortunately, the second module, U-Net, can eliminate the edge noise and make the electrical parameters of the brain tissues more accurate. As shown by the second row of Figure 6, although the structure of the human brain is complex and the contrast is high, the final imaging results are in good agreement with the real information. The model misfits and corresponding data misfits between the reconstructed results of proposed model and ground truth are shown in Table 2. It can be seen that the image enhancement module composed of U-Net can effectively reduce model misfits and data misfits of FCERNN reconstructed data.

**Figure 7:**
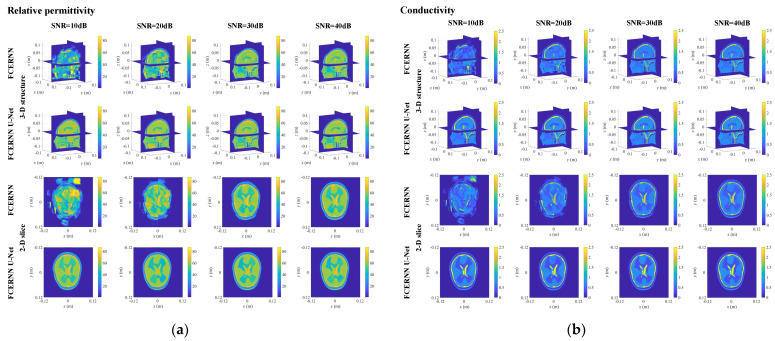
The reconstructed results obtained from the scheme under noise: (**a**) relative permittivity distribution; (**b**) conductivity distribution.

However, the measurement of scattered field will be interfered by noise in a real environment. To further evaluate the anti-noise performance of the proposed scheme in the noisy environment, different levels of white Gaussian noise are added to the measured scattered electric field. Figure 7. shows the relative permittivity and conductivity distribution of human brain reconstructed by 3D FCERNN and U-Net optimization at relative noise levels of −10 dB, −20 dB, −30 dB and −40 dB.

The results obtained from 3-D FCERNN showed that with the increasing noise level, the boundary between brain and tissues becomes more and more blurred, and more noisy blocks appear. Nevertheless, even when the relative noise level is −10 dB, the reconstructed brain tissues are almost indistinguishable. Then, after the optimization by the second module, it is not hard to see that the final imaging results are improved, and the influence of noise is improved to a certain extent.

The model misfits and data misfits of the final reconstruction results obtained by 3-D FCERNN and U-Net are listed in Table 2. It can be seen that the proposed method has some anti-noise performance in the noisy environment.

### 3.4. Case II: Detection of Anomalies

In order to evaluate the detection performance of the proposed method for anomalous scatterers, three different anomalous cases are provided which contain anomalous scatterers with random position, size and electrical parameters. 

The imaging domain of this case is discretized into 512 × 512 × 512 voxels, which is with higher imaging resolution than Case I. Hence, the number of unknowns of this case is eight times that of Case I. This is not only a great challenge for traditional inversion methods, but also difficult for machine learning-based inversion methods to handle such a high-resolution imaging task. The differences of the three abnormal scatterers test sets and the averaged training model are shown in Table 1. Three-dimensional FCERNN required approximately 18 h for training on a computer with 24 GB of video memory, and the trained model will cost 5 min during testing. U-Net cost 80 min for training and one minute for test.

**Figure 8:**
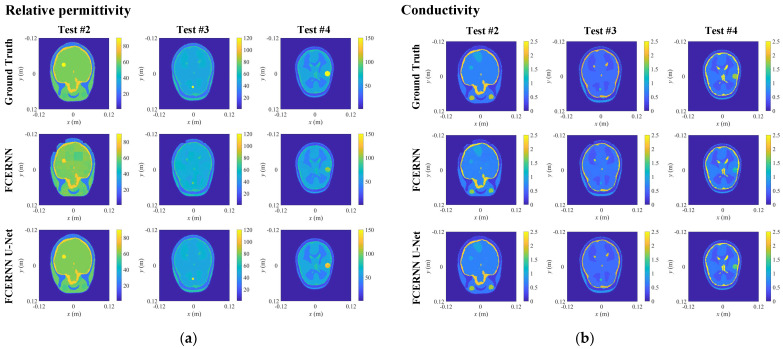
The reconstructed 2-D slices of the reconstruction results with anomalous scatterers optimized from FCERNN and U-Net: (**a**) relative permittivity distribution; (**b**) conductivity distribution.

First, Tests #2–4 are performed in an ideal noiseless environment. As shown in Figure 8, the reconstructed results of Tests #2–4 slices at *z* = −0.01, 0.006 and 0.016 planes obtained by 3-D FCERNN can approximately reconstruct the contour of abnormal scatterer and brain tissues, but the imaging accuracy is not satisfactory. Then, with the optimization by the second module, the contour, shape and electrical parameters of human brain tissues can be well imaged. Meanwhile, the position, shape, size, relative permittivity, and conductivity of each anomalous scatterer can also be imaged, even for the tiny anomalous scatterers in Test #3. The model misfits and data misfits of Tests #2–4 are listed in Table 3. It can be seen that although this numerical example is with anomalous scatterers and higher resolution, both the model misfit and data misfit are still in a satisfactory level in the noise-free environment. Moreover, the anomalous scatterers in the three cases can be reconstructed accurately, including their position, shape, size and electrical parameters.

**Figure 9:**
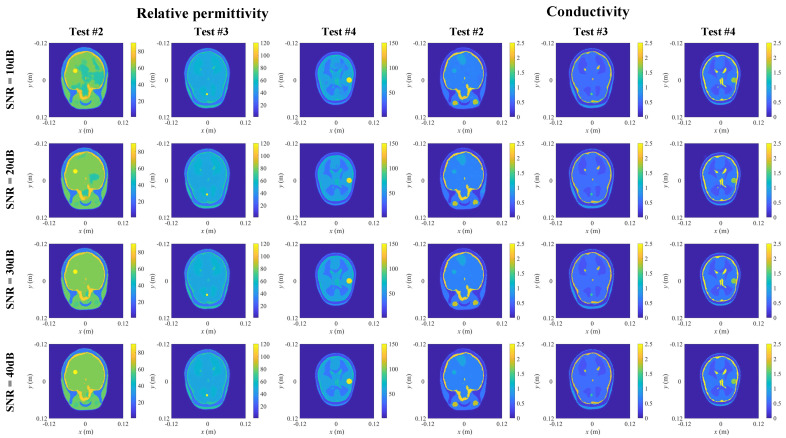
The reconstructed 2-D slices of relative permittivity and conductivity distributions obtained from the scheme with abnormal scatterer under noise.

In order to evaluate the performance of the proposed scheme in a noisy environment, white Gaussian noises with relative noise levels of −10 dB, −20 dB, −30 dB and −40 dB were added to the measured scattered electric field. The reconstructed results under those noises are shown in Figure 9. It can be seen that the proposed scheme can well reconstruct the position, size and contour of anomalous scatterers under various noises. However, at −10 dB noise level, the electrical parameters of abnormal scatterers are not accurate enough, and some noise blocks appear in brain. With the reduction in the relative noise level, this problem is improved and the imaging results becomes better and better. Both model misfits and data misfits of the reconstructed results are listed in Table 3. It can be seen that the proposed scheme can still reconstruct high-quality super-resolution human brain model in a high-noise environment, and abnormal scatterers in brain tissue can also be reconstructed accurately. Therefore, the scheme still has a good ability to detect anomalous scatterers in high noise environment.

## 4. Discussion

Three-dimensional super-resolution brain microwave imaging has great application value in clinical diagnosis and biomedical research. However, achieving super-resolution 3-D brain imaging is a very difficult task. For the existing traditional EM inversion methods, such as VBIM, the inversion performance is highly correlated with the initial values obtained by linear approximation. For the strong scatterers such as human brain, it will bring strong nonlinearity, so the linear approximation cannot provide good initial values for the traditional inversion method. In this way, in the case of strong scatterers, it is difficult to converge in the forward simulation at each iteration. 

For the machine learning based-EM inversion method, such as HNNEMIS proposed by our group, it maps the relationship between human brain scattered field and electrical parameter distribution by SJ-BPNN. SJ-BPNN discretizes 3-D objects into multiple 1-D data sequences, and each sequence is inverted by a BPNN. Thus, a large number of models are needed to train, and the imaging speed is slow and requires a large amount of computing resources. For a human brain case, HNNEMIS needs 8 h and 89 Gb for training, and 30 min and 19 Gb for testing, respectively. For this work, it needs 3 h and 24 Gb for training and 1 min and 4 Gb for testing, respectively. Thus, compared with the HNNEMIS, FCERNN can greatly reduce the training time and computing consumption.

## 5. Conclusions

The EM inverse scattering for 3-D super-resolution human brain imaging is a typical high contrast inversion task. The implementation of super-resolution introduces a huge number of unknowns, which leads to unbearable computational cost. To solve this inverse problem, this work proposes a dual-module EM inversion scheme based on a deep neural network, which includes an EM inversion module and an image enhancement module. In the EM inversion module, a 3-D FCERNN is proposed to map the measured scattered electric field to 3-D electrical properties of scatterers, such as relative permittivity and conductivity. This network has low computational cost, high accuracy and fast training speed in super-resolution 3-D imaging tasks. For the image enhancement module, U-Net is adopted to further improve the imaging quality. Moreover, to solve the collection of real human brain data, a dataset generation method based on human brain features deformation is proposed to accelerate the convergence speed of the network.

Two numerical cases are employed to verify the reliability of the proposed super-resolution 3-D brain reconstruction scheme. The first case uses a normal human brain with 256 × 256 × 256 voxels to validate the proposed scheme. The second case is employed to validate the proposed scheme for abnormal scatterer detection in human brain with 512 × 512 × 512 voxels. The inversion results of these examples in noise-free and noisy environments show that the proposed method can efficiently reconstruct the 3-D super-resolution distribution of human brain electrical properties, and has superior detection ability for human brain abnormal scatterers. Meanwhile, one should note, in this work, only one basic model is selected due to the data restriction. More basic models will make the EM inversion scheme more generalizable for human brain imaging.

## Figures and Tables

**Table 1:** Differences between the test set and the averaged training mode.

Testing Model	Normal Model		Abnormal Model	
	Test #1	Test #2	Test #3	Test #4
Model misfit				
Max model misfit (%)	84.27	104.61	95.60	73.57
Min model misfit (%)	13.98	12.02	9.42	11.04
Average model misfit (%)	56.34	66.42	57.24	58.04

**Table 2:** Model misfits and data misfits of the proposed method for normal human brain case at different noise levels.

Noise Level	3-D FCERNN		3-D FCERNN-U-Net	
	Model Misfit (%)	Data Misfit (%)	Model Misfit (%)	Data Misfit (%)
Noise Free	20.16	16.96	19.54	15.53
−10 dB	71.72	67.47	26.78	25.84
−20 dB	48.92	37.14	21.34	18.64
−30 dB	31.25	26.28	19.76	15.48
−40 dB	22.38	18.80	19.36	15.37

**Table 3:** Model misfits and data misfits of the proposed method for human brain abnormal scatterer cases at different noise levels.

Noise Level	Test	3-D FCERNN		3-D FCERNN-U-Net	
		Model Misfit (%)	Data Misfit (%)	Model Misfit (%)	Data Misfit (%)
Noise Free	Test #2	16.03	26.53	9.03	7.78
	Test #3	14.98	11.57	9.09	4.38
	Test #4	16.60	12.39	10.64	6.97
−10 dB	Test #2	47.70	71.13	16.92	33.68
	Test #3	53.40	83.56	17.24	14.91
	Test #4	60.59	136.91	19.45	16.29
−20 dB	Test #2	28.33	42.94	13.16	16.22
	Test #3	36.14	39.49	15.05	9.63
	Test #4	45.41	84.91	17.90	25.28
−30 dB	Test #2	17.94	27.65	10.36	10.10
	Test #3	21.32	23.24	11.39	7.89
	Test #4	29.87	34.07	14.64	10.19
−40 dB	Test #2	16.15	26.56	9.91	10.07
	Test #3	15.61	15.72	9.95	8.17
	Test #4	18.94	17.18	11.81	8.91

## Data Availability

Not applicable.
